# Audio-Vestibular Profile of COVID-19; Systematic Review and Meta-analysis

**DOI:** 10.22038/IJORL.2022.60404.3079

**Published:** 2022-07

**Authors:** Mehri Maleki, Mohammad Maarefvand, Ahmad Reza Nazeri, Ali Reza Akbarzadeh Baghban, Azadeh Borna

**Affiliations:** 1 *Student Research Committee, Iran University of Medical Sciences, Tehran, Iran.*; 2 *Department of Audiology, School of Rehabilitation Sciences, Iran University of Medical Sciences, Tehran, Iran. *; 3 *Department of Audiology, School of Rehabilitation Sciences, Shahid Beheshti University of Medical Sciences, Tehran, Iran.*; 4 *Proteomic Research Center Department of Biostatistics, School of Allied Medical Sciences, Shahid Beheshti University of Medical Sciences, Tehran, Iran.*

**Keywords:** Auditory, COVID19, Coronavirus, CoV-2, Ear, Hearing, Vestibular, Tinnitus, SARS

## Abstract

**Introduction:**

After more than a year of the COVID-19 pandemic, audio-vestibular problems have been reported as consequences. Several limited case report studies with different methodologies were published. This study aimed to describe the impact of COVID-19 on the auditory-vestibular system and communication problems in subjects with hearing impairment.

**Materials and Methods:**

The current systematic review was performed based on the Preferred Reporting Items for Systematic Reviews and Meta-Analysis (PRISMA) guideline. PubMed, Web of Science, and Google Scholar were searched to find relevant articles using combined keywords.

**Results:**

Out of 26 final studies, 20 studies dealt with the effects of COVID-19 on the auditory and vestibular system, and six articles examined the COVID-19 effects on hearing-impaired people and patients. In these studies, dizziness (17.8%), tinnitus (8.1%), and vertigo (2.8%) were common symptoms. Most studies were case reports (42.30%), and in terms of quality, nine studies (34.61%) were in the suitable quality group.

**Conclusions:**

COVID-19 might cause auditory-vestibular system problems by directly affecting the structures or functions of the inner ear or by weakening the immune system. The need for taking preventive measures during the COVID-19 pandemic has caused communication and social challenges, particularly for people with hearing loss.

## Introduction

After more than a year since the World Health Organization declared the coronavirus infection (COVID-19) a pandemic (1), intangible symptoms of the disease have received much attention due to reduced initial concerns about the pathogenicity of the virus, contagion, risk factors, mortality, short-term consequences (2), and methods of prevention and treatment. 

In this regard, the SARS-CoV-2 receptors detection in the CNS was of interest. Neurotological manifestations (3) and dizziness have been suggested as the most common neurological disorders caused by COVID-19 (4). 

Therefore, in addition to the evident symptoms of the disease, including fever, cough, dyspnea, chest tightness, nasal congestion, sore throat, and smell dysfunction, non-obvious symptoms such as auditory-vestibular symptoms were also considered (5).

Several case reports of sudden hearing loss, dizziness, and tinnitus during or following COVID-19 were presented, and then limited studies with different methodologies were also reported. Patient isolation is the best way to break the disease transmission chain (6). Furthermore, early identification reduces the risk of disease exacerbation and hospitalization if appropriate treatment is initiated (7). Regarding the burden of dizziness (8), tinnitus (9), vertigo (10), and hearing loss (11), identifying the physical and psychological factors that cause or exacerbate them during the pandemic seems valuable. However, social distancing and face masks on community members had psychological and lowering quality of life effects along with reduced access to audiology services in tinnitus sufferers (12-16), especially in people with hearing loss (13). Previously, two systematic review and meta-analysis studies were published to investigate audio-vestibular symptoms in COVID-19. They revealed several different results due to different search strategies and the heterogeneity of articles in terms of study design, population, and reported symptoms. This study aimed to describe the impact of COVID-19 on the audio-vestibular system besides communication problems in people with hearing impairment. In an attempt to reduce heterogeneity, the effect of mask use on people with normal hearing was not considered in this study.

## Materials and Methods

Search Protocol

The current systematic review was performed according to the guidelines of the Preferred Reporting Items for Systematic Reviews and Meta-Analysis (PRISMA) (17). 

The PubMed, Web of Science, and Google Scholar were used to find relevant articles using combined keywords "Auditory”, “ear”, “hearing”, “vestibular”, “tinnitus", “COVID-19”, “COVID19”, “SARS-CoV-2”, and/or “coronavirus" on articles published in English from 2019 to June 2021(18). As a result, a total of 618 articles were identified. 

Eligibility criteria

The target population was patients with audio-vestibular disorders and hearing impairment due to COVID-19. Only original research publications were selected. Retrospective, cohort, cross-sectional, case report, and case-control studies were included. The outcome of interest was hearing problems, tinnitus, disequilibrium, vertigo, dizziness, and other audio-vestibular-related problems. Articles written in languages other than English, with study design such as reviews, letters to editors, editorials, and studies without an available full text were excluded.

***Data selection and management process***

The final papers were assessed through a three-phase screening procedure: Title, abstract, and full-text screening. First, two independent reviewers primarily screened and examined the articles in each phase and omitted unrelated cases based on the inclusion and exclusion criteria. In the cases of ambiguity, first, the abstracts and then the full texts were investigated. All three phases were individually directed by two reviewers (M.M. and B.A.). 

**Fig 1:**
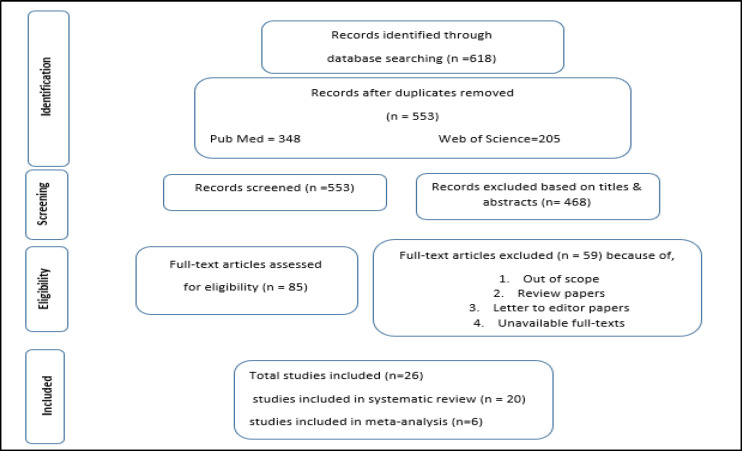
Flow diagram of review process based on the Preferred Items for Systematic R eviews and Meta-Analyses

If there was any discrepancy, the reviewers argued to reach an agreement. Eighty-five retrieved articles during the first and second phases and 26 in the third step remained for further review. The following data were extracted from all the eligible studies using a pre-design data extraction sheet, including Author(s), publication year, assessment method, study design, participant demographics, and outcomes. The screening process and results were summarized in a PRISMA flow diagram (Figure 1).

***Risk of bias in individual studies***

The risk of bias was assessed by two researchers using the National Institutes of Health’s (NIH) quality assessment tools, which were designed to evaluate the risk of bias in epidemiological observational studies and many other types (NIH National Heart, Lung, and Blood Institute 2014) (19). 

These tools provide a different checklist for each study design (e.g., Quality Assessment Tool for Case Series Studies and Controlled Intervention Studies). The quality of each study was rated as either good, fair, or poor. The overall rating of reviewers was reported (Table 1).

***Data synthesis***

The extracted data were analyzed using Stata/SE 14 for Windows (Stata Corp LP). We used the random meta-analysis model of weighted inverse variances to obtain an overall summary assessment of the prevalence across studies. 

The I^2^ statistics measured the heterogeneity of the studies, and the bias in publication for tinnitus was assessed using Egger’s linear regression test. 

## Results

*Search and selection of studies*

We primarily recognized more than 618 records, of which 65 were duplicates and deleted. The titles and abstracts of the remaining 553 records were screened against the inclusion criteria. Of them, 85 met the eligibility criteria. In full-text screening only 26 articles had eligibility. The screening process and results are shown in a PRISMA flow diagram (Figure 1).

***Characteristics of the included studies***

Out of 26 final studies, 20 studies dealt with the effects of COVID-19 on the auditory and vestibular system (of which six articles were meta-analyzed with the sample size criterion (mark with an asterisk in table 1)), and six publications examined the effects of COVID-19 on hearing-impaired people (Table 1). 

Based on pooled estimates, dizziness (17.8%) and tinnitus (8.1%) were common symptoms in these studies. Symptoms such as otalgia, fullness, instability, auditory hallucinations, and Bell's palsy were also reported. Among the selected studies, most studies were case reports (42.30%), and in terms of quality, nine studies (34.61%) were in the good quality group.

**Table 1:** Included articles in systematic review and meta- analysis (SSNHL; Sudden Sensory-Neural Hearing Loss, HHIA; Hearing Handicap Inventory, THI; tinnitus Handicap Inventory, TEOAE; Transient Evoked Otoacoustic Emission, VEMP; Vestibular Evoked Myogenic Potential, HFPTT; High Frequency Pure-Tone Thresholds, HL; Hearing Loss, CI; Cochlear Implant)

Studies reported auditory -vestibular effects of COVID-19						
**Authors**	**Location**	**Participant (s)**	**Design**	**Method**	**Outcomes**	**Quality Assessment**
Alexander Chern, et al (2021) (20)	New York	18 year	Case Report	Audiometry Tympanometry	Bilateral SSNHL,Fullness, Vertigo, Nausea, Vomiting	Fair
Clouden (2020)(70)	. -	46 year	Case Report	Imaging	Auditory and visual hallucinations	Poor
Elibol (2020)(37)*	Turkey	155	Retrospective	Medical files	Otalgia 2% Tinnitus 1.2% Bell's palsy 0.6% SSNHL 0.6%	Good
Elkhaled, et al (2020) (71)	Qatar	23 year	Case Report	Imaging	Auditory hallucinations	Poor
Fidan (2020) (72)	Turkey	35	Case Report	Audiometry Tympanometry	Otalgia Otitis Media Tinnitus	Poor
Frenia (2020)(73)	Italy	50	Cross Sectional	HHIA THI	Auditory discomfort 40 % Tinnitus 20 %	Fair
Kilic, et al (2020)(24)	Turkey	5	Case Report	Audiometry	Unilateral SSNHL 20 %	Fair
ÖzçelikKorkmaz,et al (2020)(6)*	Turkey	116	Cohort	Questionnaire	Dizziness 31.8% Tinnitus 11% True Vertigo 6% Hearing Impairment 5.1%	Fair
Koumpa, et al (2020) (21)	England	45 year	Case Report	Audiometry		Fair
Lamounier, et al (2020) (22)	Brazil	67 year	Case Report	Audiometry	SSNHL	Good
Lang, et al (2020) (29)	Ireland	30 year	Case Report	Audiometry Imaging	SSNHL and Tinnitus	Fair
Lechien, et al (2020) (5)*	Europe	1,420	Cross-Sectional	Questionnaire	Rotatory Vertigo 0.42 % Tinnitus 0.35 %	Good
Liang, et al (2020) (38)*	China	86	Cross Sectional	Questionnaire	Tinnitus 3.5 %	Fair
Malayala& Raza (2020) (46)	USA	29 year	Case Report		Vestibular Neuronitis	Good
Micarelli, et al (2020) (39)*	Italy	1380	Cross Sectional	Online survey	Dizziness 6.2% Disequilibrium 6.3% Tinnitus 10.4% Fullness 8.6%	Fair
Miri,et al (2020) (74)	Iran	2	Case Report			Fair
Mustafa (2020) (75)	Egypt	20	Case- Control	TEOAE VEMP	HFPTT and TEOAE amplitudes worsened	Poor
Raad, et al (2020)(23)	Iran	8	Case Series	Otoscopy Tympanometry Audiometry	Otalgia 75% Hearing Loss 87.5 % Otitis Media 37.5 % Acute Otitis Media with perforation 12.5 %	Fair
Vanaparthy, et al (2020) (47)	United States	63 year,	Case Report	Physical Examination	dizziness Unsteady gait Vertigo Vestibular Neuritis	Good
Viola, et al (2020) (36)*	Italy	185	Cross Sectional	Questionnaire	Equilibrium disorders 18.4% Dizziness 94.1% Vertigo 5.9% Tinnitus 23.2% Tinnitus And Equilibrium 7.6%	Fair
Studies Reported Communication Problems Of Hearing Impaired People In COVID-19 Era						
Alqudah, et al (2021) (76)	Jordan	278 Individuals with hearing loss	Cross-Sectional	Questionnaire	Hearing difficulties 47.5 % Ear problems such as ear ringing, ear infections, vertigo 70.5 % Communication problems 56.8 % Deterioration in academic or work performance 21.9%	Good
Alsadoon&Turkestani (2020) (77)	Saudi arabia	11 instructors	Qualitative	Unstructured phone interviews	Time, technical problems, and lack of simultaneous translation	Fair
Ariapooran&Khezeli (2021) (67)	Iran	56 Individuals with hearing loss	Cross Sectional	Self-report	Anxiety disorders in HL group 37.5% (Higher in deaf than normal adolescent)	Fair
Dunn, et al (2020)(69)	USA	48 adults with cochlear implant	Retrospective	Smartphone app Questionnaires	CI recipients felt less socially isolated and less anxiety during COVID in comparison to pre-COVID	Good
Yang, et al (2021)(68)	China	420 students with hearing loss 800 normal hearing students	Observational	Questionnaire	Hearing loss students are frustrated and vulnerable to stress symptoms	Good
Saunders, et al (2020) (13)	UK	460	Survey	Questionnaire	Face coverings have negative impacts on communication especially in people with hearing loss	Good

***Meta-analysis results***

**Fig 2:**
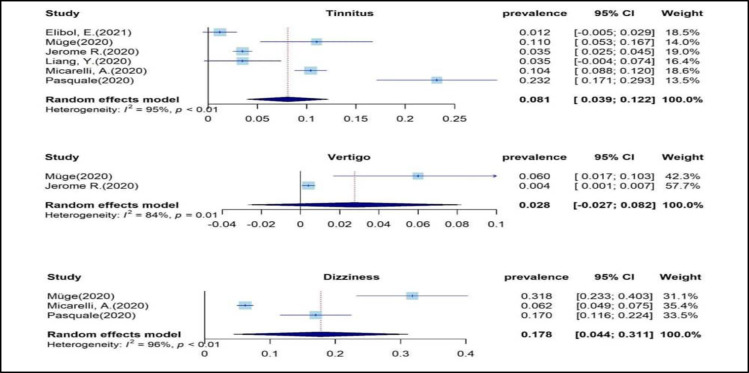
forest plot for the event rate tinnitus, vertigo and dizziness in six studies. The square size indicates the statistical weight for each study. The horizontal line represents 95% CI, and the diamond summarizes the overall pooled estimate of event rate and its corresponding 95% CI. CI, confidence interval

Of 20 articles, nine articles reported *tinnitus*. Six of them were included in the meta-analysis. The *I*^2^statistic showed that the studies were significantly heterogeneous (I^2 ^= 95.5 %, P<0.001), so the random-effects analysis was used (figure 2). Accordingly, the estimated pooled prevalence of tinnitus was 8.1% (95% CI: 3.9-12.2%). The heterogeneity assessment of six studies that reported data on tinnitus is shown in Figure 2. The Egger test was performed to investigate the publication bias, which showed no publication bias (p=0.312). Nnine were about. Four were about *dizziness*, and three were included in the meta-analysis. The I^2^ statistic showed that the studies were significantly heterogeneous (I^2^=94.2 %, P<0.001), so the random-effects analysis was used (figure 2). Three publications were used to estimate dizziness prevalence with a total of 1,681 COVID-19 patients. Accordingly, the estimated pooled prevalence of dizziness was 17.8% (95% CI: 4.4-31.1%). The heterogeneity assessment of the three studies that reported data on dizziness is shown in Figure 2, and five articles concerned ***vertigo***, and only were two studies used in the meta-analysis. The I^2^ statistic showed that the studies were significantly heterogeneous (I^2^= 84.4 %, P=0.01), so the random-effects analysis was used (figure 2). 

Two publications were used for the estimation of vertigo prevalence with a total of 1,536 COVID-19 patients. Accordingly, the estimated pooled prevalence of vertigo was 2.8% (95% CI: 0-8.2%). The heterogeneity assessment of the two studies that reported data on vertigo is shown in Figure 2.

## Discussion

This systematic review and meta-analysis aimed to provide evidence for COVID-19 effects on audio-vestibular problems. 

***Hearing loss in COVID-19 patients:***

In nine articles, hearing loss (Mostly sudden sensorineural hearing loss) was considered as a symptom or complication of COVID-19 (Table 1) (20-22) either as a conductive hearing loss in a case-series study (23) or sudden sensory neural hearing loss in a cross-sectional study (24). The incidence of hearing loss following viral infection was unprecedented (25) after congenital or acquired hearing loss has been reported in hepatitis (26), measles, mumps, meningitis, rubella, and HIV (25). Peripheral or central auditory dysfunction after viral diseases may occur due to damage to the inner ears (25) or auditory brainstem (27). It may be the consequence of host-immune response due to hair cell or stereovascularis damage (26) or vasculitis (28) and a cause of hearing loss following hepatitis (25), possibly due to cochlear inflammation and perilymphatic tissues (29), and the stress response of inner ear antigens against viral infections and neuritis (30). Cochlear inflammation can cause sudden sensorineural hearing loss following an inflammatory response and increased cytokines due to coronavirus infection (21). Also, according to the reports of the presence of Angiotensin-converting Enzyme2 (ACE2) as a coronavirus receptor in glial cells and neurons in explaining COVID-19-induced neurological involvement (3,31), its effect on auditory-vestibular nerves seems possible (6). 

Therefore, there is a possibility of hearing loss in COVID-19 patients (24). Hearing loss was reported as the only or first sign of disease in some studies (23,24), so it is necessary to prevent the progression of the disease and pulmonary involvement. The possibility of COVID-19 in patients with sudden hearing loss, which requires corticosteroid therapy in the first 72 hours, to increase the chance of threshold recovery (32). However, due to the small number of studies in this systematic review, the use of questionnaire methods and the lack of audiological assessments possibly lead to the misdiagnosis of low-threshold changes, lack of reliable knowledge on the type and degree of hearing loss, lack of questionnaire methods, and a control group in the studies, we must be cautious in the interpretation of the results. Two studies reported a hearing loss as the first or only symptom of COVID-19 (23,24), while five studies reported it following COVID-19, which in three of them was associated with hospitalization (20-22). 

Conductive hearing loss was reported in one study (23), but the others reported sudden sensorineural hearing loss. Differences in onset, type, and degree of hearing loss after ototoxic medications in early COVID-19 treatment protocols that may contribute to hearing loss (33) demonstrate the necessity of caution in interpretation.

***Tinnitus in COVID-19 patients:***

Mild to debilitating tinnitus with a prevalence of 7.1 to 14.6% may appear (34). The pathological mechanism of tinnitus is still unknown. It may be due to dysfunction in different parts of the auditory pathway from the external ear to the auditory cortex (35). Therefore, due to the possible involvement of the peripheral and central auditory system in COVID-19, it seems that tinnitus occurs following COVID-19. 

There is evidence indicating the genome sequence of the coronavirus in the brain and observing pathological changes in the hypothalamus and cortex (36), leading to the probability of neuroatological disorders such as tinnitus. In addition, the occurrence of tinnitus following vascular disorders for which there is evidence of COVID-19 can be considered a cause of tinnitus following infection (36), which is confirmed by the reports of tinnitus in other types of coronavirus diseases (27). Our meta-analysis on six papers (5,6,36-39) (figure 2) demonstrated the occurrence rate of 8.1% (95% CI: 3.9-12.2%) for tinnitus in patients with COVID-19. This value is in the lower limit reported by Almufarrij et al. (95% CI: 6.3-26.1%). This finding may be due to differences in the inclusion criteria for including articles in the meta-analyses. In the study by Almufarrij et al. (26), all studies related to tinnitus were included, while in the present study, only studies that reported the appearance of tinnitus following COVID-19 were considered. 

Despite the differences in databases and search strategies, since the Eger analysis showed no publication bias, the occurrence rate reported in the present meta-analysis was in agreement with the Jafari et al. study (95% CI: 12-15.3%) (7). Moreover, studies demonstrated that COVID-19 could increase tinnitus severity (14, 15,40). Restrict social interactions and increased anxiety during the lockdown period might be among the causes of this oppressive experience (14).

***Dizziness/vertigo in COVID-19 patients:***

Dizziness is a common complaint in primary care that affects almost everyone throughout their lives. International studies express that dizziness and vertigo have a significant burden on the public (8,10,41). Dizziness is a general term identified as one of four types: vertigo, disequilibrium, impending faint, and vague lightheadedness (42). Vertigo is a sign of vestibular dysfunction (peripheral or central), described as a sense of rotation (42,43). Nevertheless, most dizzy patients belong to disequilibrium, impending faint, and vague lightheadedness (non-vestibular system disorders) (42). A systematic review of 20 large population-based studies in 2014 demonstrated a lifetime prevalence of dizziness between 17 and 30% and vertigo between 3 and 10% (44). 

***Dizziness*** *:*

The present meta-analysis of three papers (6, 36, 39) (figure 2) demonstrated the occurrence rate of 17.8% (95% CI: 4.4-31.1%) for dizziness after COVID-19. As dizziness is a non-specific term (43, 45), in this study, contrary to a previous investigation, articles related to vertigo and dizziness were analyzed separately. Therefore, the high prevalence of dizziness in the included reports was within the upper limit reported by Jafari et al. (95% CI: 6.3-26.1%) (7). 

***Vertigo:***

Vertigo was reported as a complication of COVID-19 in five studies (table 1). The meta-analysis of two studies (5,6) (Figure 2) with cohort and cross-sectional design (n = 1536) showed an occurrence rate of 2.8% (95% CI: 0-8.2-95%). This value is in the lower limit of Almufarrij et al. (95% CI: 0.01-26.4%) (26). Different inclusion criteria were the source of difference between Almufarrij et al. (26) and this study. They mentioned that by excluding studies with both vertigo and dizziness, the pooled estimate was reduced to 3.4%, consistent with the present meta-analysis results. In our included studies, vertigo was assessed by questionnaires or questions. Vestibular neuritis was reported in the two of the three case studies (46,47). Vestibular neuritis is an inflammation of the auditory-atrial nerve with a manifestation of vertigo, nausea, and gait imbalance (48). The effects of SARS-CoV-2 on nervous tissue can be due to the infection of the central nervous system itself or vascular damage caused by vasculitis or vasculopathy, similar to the mechanism of Varicella-Zoster Virus (VZV) and Human Immunodeficiency Virus (HIV) (36,49,50). Vasculitis is usually associated with audio-vestibular symptoms, and primary cardiovascular disease can induce dizziness (36,51,52). Also, BPPV was the most common peripheral vestibular disorder after COVID-19 (53). Vestibular neuritis, Meniere disease, and sudden hearing loss are the known causes of BPPV (54). In addition, hospitalization and prolonged rest after an illness can provoke BPPV (55). It seems vestibular rehabilitation can be effective in the COVID-19 management induced vestibular neuritis and BPPV like other vestibular dysfunctions (56). Considering the heterogeneity between studies (7,26), and that none of the studies used standard questionnaires, balance and behavioral and laboratory tests, and the information obtained by asking questions online or in person, the results should be interpreted with caution.

***Communication problems after hearing loss during the COVID-19:***

Out of 30, six papers addressed this issue. Given that speech recognition is an audiovisual phenomenon (57), the need to take preventive measures (using masks and social distancing during the pandemic) has made communication challenging, particularly for people with hearing loss (13). Speech and lip-reading and the interaction between visual and auditory senses, known as the McGurk effect, contribute to understanding speech, especially in noise or poor conditions (57,58). Thus, responses to the audiovisual stimulus are affected by auditory and visual abilities and audiovisual integration capabilities (57). Physical distance can reduce the signal-to-noise ratio, especially in difficult situations, and mask use can decrease the quality of speech signals. In Magee et al.’s study (59) that investigated the effects of mask use on speech comprehension, further attenuation of frequencies higher than 3 kHz, which play a central role in speech, compared to low frequencies, was raised. 

Also, another study showed a significant decrease in speech comprehension in noise and an auditory effort using masks. In this study, the reductions of frequencies above 1 kHz and no changes in frequencies below 1 kHz were proposed (60). Therefore, it seems that a lower signal-to-noise ratio in the high-frequency range can lead to a decreased speech perception (59,60), which will be more visible in strict listening conditions, hearing loss, and the elderly (61).

These problems with the lack of required visual cues for understanding speech in noise (62) challenge audiovisual performance. It can affect the ability to respond to audiovisual stimuli and the McGurk effect and increase the likelihood of multisensory errors. Since McGurk's impact is more induced in hearing-impaired people than in normals (63), this may partly explain the adverse effects of COVID-19 on the social communication of hearing-impaired people. In addition, using a mask can affect the nonverbal part of communication. It could be the mirror neuron system. Mirror neurons are activated when you respond to an unknown person's smile with a smile. Mirror neurons explain some social behaviors, like mind-reading and imitation (64), which may become inaccessible due to mask use and lack of access to facial expressions. Mirror neurons and their roles in rehabilitation (65,66), especially in hearing-impairment subjects, are of interest. 

Therefore, the reduced information from the mirror neuron system after using the mask might lead to communication problems and disruption of auditory rehabilitation of people with hearing loss during the pandemic. Communication problems reported in studies during the COVID-19 pandemic can have psychological manifestations such as anxiety for the hearing impaired. A cross-sectional study considering 56 adolescents with hearing loss showed a high rate of anxiety in these children (37.5%) compared to healthy people (67). Also, Yang et al., in a questionnaire study on stress symptoms in 420 students with hearing loss reported that these people were more vulnerable than healthy subjects (68). However, Camille et al., in testing 48 cochlear implant users, reported less social isolation and anxiety before the COVID-19 pandemic. The discrepancy might be due to the relaxing listening experiences in a controlled environment with fewer speakers (69).

This review has several limitations, primarily due to the high diversity in article design, limited studies with sufficient sample size and well-designed, and limited studies that used standard audiology tests for auditory and vestibular assessments. Another limitation has been the inclusion of only two vertigo articles in the meta-analysis. To draw the reader’s attention to the clinical significance of the separate study of vertigo and dizziness following Covid19, the authors have done it with the knowledge of a low statistical validity, so the results should be interpreted with caution. Studies with larger sample sizes, using more robust designs and standard tools seem necessary. Further studies are suggested to evaluate the long-term effects of COVID-19 on the central auditory nervous system, the impact of medical treatment on the appearance of audio-vestibular symptoms such as tinnitus, and the influence of hearing and communication deprivation on groups with special needs.

## Conclusion

The exact mechanism of COVID-19 on the audio-vestibular system is not known. Coronavirus may cause the symptoms due to impairment to structures or immune system related to functions of the inner ear, increasing the likelihood of these symptoms. As there is a limited period for the development of audiovisual integration in children, due to the prolongation of the pandemic, hearing-impaired children might be deprived of this ability by practicing in everyday living environments and miss the main period of audiovisual integration development. It is also substantial to pay attention to the communication problems of hearing-impaired people and provide communication facilitation strategies such as using transparent masks, captioning, and hearing aids re-fitting along with training methods and tele-rehabilitation during this pandemic.Therefore, it seems that the implementation of systematic studies to investigate the impact of COVID-19 on the audio-vestibular system and also the long-term effects of the pandemic on the peripheral auditory system and central auditory nervous system alongside the pandemic effects on hearing impairment and utility rehabilitation service is essential in the long run.
